# Looking Beyond Nutrients, How to Assess Diet Quality in an Inflammatory Bowel Disease Population—A Narrative Review

**DOI:** 10.3390/nu17142343

**Published:** 2025-07-17

**Authors:** Laura J. Portmann, Jessica A. Fitzpatrick, Emma P. Halmos, Robert V. Bryant, Alice S. Day

**Affiliations:** 1 Inflammatory Bowel Disease Service, Department of Gastroenterology, The Queen Elizabeth Hospital, Woodville South, SA 5011, Australia; 2 Inflammatory Bowel Disease Research Group, The Basil Hetzel Institute for Translational Health, Woodville South, SA 5011, Australia; 3 Department of Gastroenterology, Monash University, Melbourne, VIC 3004, Australia; 4 Department of Gastroenterology, Alfred Health, Melbourne, VIC 3004, Australia; 5 School of Medicine, Faculty of Health and Medical Sciences, The University of Adelaide, Adelaide, SA 5000, Australia

**Keywords:** inflammatory bowel disease, diet quality, diet quality index, dietary assessment, dietary patterns

## Abstract

**Background**: Dietary assessment in inflammatory bowel disease (IBD) is moving away from individual food and nutrient analyses and towards dietary patterns (e.g., Mediterranean diet, Western diet) and diet quality assessment that are increasingly implicated in IBD onset and course. However, an IBD-specific diet quality index (DQI) does not exist. This review aimed to identify current DQIs and assess their suitability for an IBD population. **Methods**: MEDLINE and EmCare databases were systematically searched for a-priori, food-based DQI reflecting current dietary guidelines and/or nutrition science. Data extracted were adapted from optimal DQI criteria, including quality measures of adequacy, moderation, variety and balance and DQI evaluation. **Results**: Twenty-four DQI were identified. No DQI included all optimal DQI criteria. The Dietary Guideline Index 2013 (DGI-2013) most closely met the criteria, followed by the Dutch Healthy Diet Index-2015 (DHD-Index 2015), Planetary Health Diet Index (PHDI) and Healthy Eating Index for Australian Adults-2013 (HEIFA-2013). Most DQI assessed adequacy (22/24, 92%) and moderation (21/24, 88%), half assessed balance (12/24) while few assessed variety (8/24, 33%). Application of other optimal DQI criteria varied. Food frequency questionnaire (13/24) and 24 h diet recall (12/24) were the most common dietary assessment methods used. Most DQI (17/24, 71%) were validated; however, not for an IBD population. Few were evaluated for reliability (6/24) or reproducibility (1/24). **Conclusions**: No DQI meets all optimal criteria for an IBD-specific DQI. The DGI-2013 met the most criteria, followed by the DHD Index-2015, PHDI and HEIFA-2013 and may be most appropriate for an IBD population. An IBD-specific DQI is lacking and needed.

## 1. Introduction

Dietary research in inflammatory bowel disease (IBD) remains a complex and emerging area. Many large prospective cohort studies have investigated associations between specific nutrient and food components with onset and incidence of Crohn’s disease and ulcerative colitis [1,2]. These studies typically describe diet in macro- and micronutrient and/or food group composition, usually gathered from food frequency questionnaires (FFQ). This aligns with current nutrient-focused dietary recommendations for IBD, with none providing specific food-based recommendations and typically encourage dietary intakes in line with healthy eating dietary guidelines globally [3,4,5].

However, emerging evidence suggests specific dietary patterns such as the Western diet or Mediterranean diet (MED) may exert unfavourable or protective effects on IBD pathogenesis and disease course rather than individual nutrients or dietary components alone [6,7,8,9]. Dietary patterns of higher diet quality have even been associated with reduced gastrointestinal inflammatory markers in a healthy population [10]. Another approach of interest involves exploring degrees of food processing, specifically classifying ultra-processed food (UPF), such as application of the NOVA classification system [11]. This method is commonly used as a pseudo-marker for general food quality but is fraught with inaccuracies, with carefully designed diets for therapeutic trials based on healthy eating dietary guidelines still able to comprise proportions of UPF that account for up to 70% of energy intake [12].

Hence, dietary pattern assessment that can comprehensively consider diet quality and the complexity of interactions between foods and nutrients may better predict health outcomes [13]. A diet quality index (DQI) is an a-priori scoring tool that holistically evaluates dietary patterns across four dimensions of adequacy, moderation, variety and balance, defined by national dietary guidelines or commonly described dietary patterns (e.g., MED) [14,15]. Burggraf et al. has further defined optimal construction criteria for a DQI including recommendations for its theoretical framework, component structure, scoring systems and evaluation processes [14].

While appropriate DQIs to apply to healthy populations have been identified [16,17], an IBD-specific DQI does not exist. It is currently unclear which DQI is most appropriate to use in an IBD population to assess disease association or predict response to therapeutic diets or clinical outcomes. To our knowledge, only one systematic review has assessed habitual diet using DQIs (e.g., Mediterranean Diet Score, Dietary Inflammatory Index) and IBD risk, progression and disease activity in longitudinal cohort and observational case–control studies but did not evaluate the applicability or appropriateness of DQIs used [18].

To advance dietary research and gain deeper understanding of IBD development and prevention beyond nutrient intake, it is important to find an accurate and suitable DQI that can be applied to therapeutic diets for IBD to help understand their effectiveness, informing development of successful therapeutic dietary strategies for IBD. This narrative literature review aims to identify current DQIs and assess their suitability for use on an IBD population.

## 2. Materials and Methods

### 2.1. Search Strategy

Electronic databases, MEDLINE and EmCare were searched until 16 May 2025 using search terms including “diet*”, “healthy eating pattern”, “nutri*”, “food”, “index”, “indic*”, “score”, “tool”, “metric*”, “quality”, “inflammat*” (see Supplementary Table S1 for full search strategy). Articles were limited to those published between 2013 and 2025, reflecting the publishing dates of current national dietary guidelines [19,20,21,22,23], in humans and available in English.

### 2.2. Eligibility Criteria

Included studies were original articles of the most current developed or updated version of an a priori, food-based DQI that reflected current national dietary guidelines and/or latest nutritional science. Articles were excluded if DQI were posteriori, nutrient-only, developed for specific non-IBD population groups (e.g., children, pregnant women, athletes) or diseases (e.g., cardiovascular disease, diabetes), or not applied to the individual level (e.g., collective household intake, food industry). See Supplementary Table S2 for full criteria.

### 2.3. Screening and Data Synthesis

**Figure 1:**
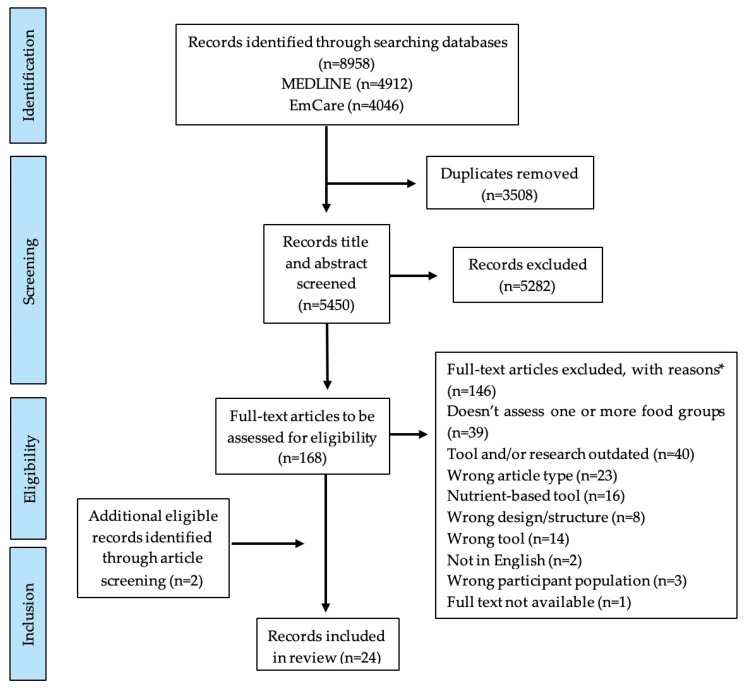
PRISMA flow diagram which details review selection processes for articles eligible for this narrative review assessing current diet quality indices and their applicability to an inflammatory bowel disease population. **Footnotes.** * categories of exclusion reasons as listed.

After duplicate removal, article titles and abstracts were screened by a single reviewer (LJP). Eligible articles were full text screened using inclusion and exclusion criteria by a single reviewer (LJP). If eligibility was unclear, the paper was discussed with a second reviewer (ASD) to reach consensus. Where an article was not the primary article for a DQI, the primary reference was obtained and screened for eligibility. Reference lists of similar existing DQI literature reviews were screened for further eligible articles [16,17,18]. Figure 1 outlines the PRISMA flow diagram for article inclusion and exclusion in this review. For all included articles, individual searches using Google Scholar and the articles’ citations were conducted to determine if a DQI had undergone additional validation studies, and/or had been used to evaluate diet quality in an IBD population.

### 2.4. Data Extraction and Quality Assessment

Data were extracted from included articles using a tool designed specifically for this review, adapted from optimal DQI criteria described by Burggraf et al. [14]. These defined criteria have not been validated or formalised into a specific assessment or quality appraisal tool; however, they were developed from expert review of international DQI and guided by the Organisation for Economic Cooperation and Development Handbook on Constructing Composite Indicators [24]. Adaptations included assessment against current dietary recommendations for IBD, being inclusion of at minimum all five key food groups (fruits, vegetables, grains, dairy products and animal- and plant-based proteins) comprising population dietary guidelines. Information extracted included the DQI’s development framework, completeness of diet quality measures of adequacy, moderation, variety and balance assessed for food components, its scoring framework and any evaluation of the DQI, including overall and gut-specific health outcomes it has been applied to assess.

## 3. Results

Twenty-four articles describing 24 DQIs applicable to adults with IBD were identified [25,26,27,28,29,30,31,32,33,34,35,36,37,38,39,40,41,42,43,44,45,46,47,48] (Figure 1). Table 1 outlines the study details and theoretical framework of the identified DQIs. Included articles were published between 2014 and 2025 with the majority from Australia (*n* = 7) [26,31,33,34,35,42,44], North America (*n* = 5) [27,36,38,41,43], Asia (*n* = 5) [39,45,46,47,48] and Europe (*n* = 4) [30,32,37,40], as well as one developed from an international cohort [28].

Sixteen DQIs were adapted from existing DQI or referenced other dietary scoring tools for development [25,26,27,28,30,31,35,36,37,38,39,40,43,44,47,48] (Table 1). Population-based dietary guidelines were used to develop 19/24 (79%) DQIs [25,26,27,30,31,32,33,34,35,38,39,40,41,42,43,44,46,47,48]. Of these, seven also used existing literature on defined dietary patterns (e.g., MED, World Health Organisation recommendations) [26,27,32,34,41,43,47]. The remaining five DQIs were developed from defined dietary patterns alone [28,29,36,37,45].

### 3.1. Optimal Criteria Components for an IBD-Specific DQI

In Table 2, the key construct components for included DQIs are outlined.

### 3.2. Dimensions

As Table 2 depicts, across DQI, assessment of the four key dimensions varied. None sufficiently assessed all four dimensions. Most DQIs assessed adequacy (22/24, 92%) [25,26,28,29,30,31,33,34,35,36,37,38,39,40,41,42,43,44,45,46,47,48] and moderation (21/24, 88%) [25,26,28,29,30,31,33,34,36,37,38,39,40,41,42,43,44,45,46,47,48]. Half (12/24) [26,27,29,30,33,38,39,40,41,42,43,44] at least partially assessed balance, while eight (33%) [28,31,32,35,38,42,44,47] at least partially assessed variety.

All DQIs assessed whole food groups reflective of population-based dietary guidelines (e.g., fruits, vegetables, grains, dairy products and animal- and plant-based proteins); however, there was heterogeneity for food and nutrient components that were assessed. For example, Roy et al. [42] assessed “total vegetable” intake whereas Bromage et al. [28] assessed vegetables as “dark leafy green vegetables”, “cruciferous vegetables”, “deep orange vegetables” and “other vegetables” categories. Supplementary Table S3 details the food group and nutrient components included within each DQI. Ultra-processed foods were assessed in 16/24 (67%) DQI [25,26,28,30,33,34,35,36,39,40,41,42,43,44,46,48].

#### 3.2.1. Adequacy and Moderation

While most DQIs assessed adequacy (92%), referring to encouraged dietary components perceived as beneficial to health and moderation (88%), referring to dietary components recommended to limit perceived as adverse to health, there was variation in whether food group, food and nutrient components were considered in adequacy and/or moderation dimensions (see Supplementary Table S3). For example, 11/24 (46%) DQIs [25,28,33,35,38,39,42,43,44,46,48] included red meats within the adequacy dimension; conversely, 10/24 (42%) [26,29,30,31,36,37,40,41,45,47] included red meats in the moderation dimension and one (4%) [34] included red meats in both adequacy and moderation dimensions.

#### 3.2.2. Variety

Of eight (33%) DQIs [28,31,32,35,38,42,44,47] assessing the variety dimension, five [28,38,42,44,47] only partially assessed this dimension, measuring variety only within one or two food groups (e.g., only types of fruit and vegetables) (see Supplementary Table S3). The Dietary Diversity Score (DDS) was the only DQI that solely assessed the variety dimension [32].

#### 3.2.3. Balance

Of twelve (50%) DQIs [26,27,29,30,33,38,39,40,41,42,43,44] assessing the balance dimension, eight only partially assessed this dimension, comparing the balance of only one or two foods or nutrients. Common components assessed included ratio of saturated, unsaturated and/or total fats to total energy (*n* = 8) [27,30,33,38,39,41,42,43], whole grains to total grains (*n* = 4) [27,38,40,44], added sugars to total energy (*n* = 5) [27,30,41,42,43] and water to total beverages (*n* = 4) [26,27,42,44] (see Supplementary Table S3). The Healthy Eating Food Index 2019 (HEFI-2019) [27] was the only DQI which solely assessed the balance dimension across all food groups.

### 3.3. Other DQI Construction Components

Shown in Table 2, most DQIs (23/24, 96%) [25,26,27,28,29,30,31,32,33,34,35,36,37,38,39,40,41,42,44,45,46,47,48] were applied to retrospective methods of dietary assessment, specifically FFQ (*n* = 13) [26,28,29,31,32,33,35,37,39,40,42,44,48], 24 h diet recalls (*n* = 12) [25,27,28,30,34,36,38,40,41,45,46,47] and/or dietitian-collected diet history (*n* = 1) [34]. Only one DQI was applied to prospective methods (weighed food records) [42] and one did not specify [43]. Five DQIs [27,31,32,33,35] were developed specifically for the respective diet assessment method they were applied to.

Scoring structures and methods varied across DQIs. Two-thirds of (16/24, 67%) DQIs [25,27,29,35,36,37,38,39,40,41,43,44,45,46,47,48] used the recommended metric scoring system and all except one [31] had scoring cut-off points. Most DQIs (21/24, 88%) [25,26,27,29,30,33,34,35,36,37,38,39,40,41,42,43,44,45,46,47,48] used recommended normative cut-offs, that reflect evidence-derived recommendations (e.g., nutrient reference values, guideline serving quantities) rather than percentile cut-offs derived from study populations (e.g., quartile intakes). However, only 10/21 (48%) DQIs [26,29,33,35,39,40,41,42,43,44] using normative cut-offs were group-specific, accounting for age and gender dietary intake recommendation differences.

#### Aggregation and Evaluation of DQIs

Diet quality indices utilised differing valuation approaches. No DQI used only non-linear valuation, referring to minimum and maximum intake thresholds for scoring, as recommended to account for foods and nutrients with both associated health benefits and risks rather than linear, one directional scoring [14]. A total of 11 of 24 (46%) DQIs [25,28,29,30,33,34,38,40,45,46,48] used a combination of linear and non-linear valuation for various components based on existing diet-disease relationship knowledge. However, non-linear valuation was not used consistently across a particular food, food group or nutrient. For example, ten DQIs [26,31,35,36,39,41,42,43,44,47] valuated the dairy food group a positive linear score where increasing intake increases points allocated, one [37] valuated dairy a negative linear score where increasing intake decreases points allocated and nine [25,29,30,33,38,40,45,46,48] valuated dairy a non-linear score, where intakes both below or above a certain range decreased points allocated (see Supplementary Table S3).

Only 10/24 (42%) DQIs [27,28,29,30,33,39,41,42,47,48] used unequal weighting across included DQI components as recommended to account for different weighted contributions of foods and nutrients to established health and disease outcomes, where intake of one particular food, food group or nutrient may have more impact on health than another (Table 2). For example, the HEFI-2019 assigns a maximum 20 points to total fruits and vegetables, but only a maximum 5 points to total protein foods [27].

Broadly, 17/24 (71%) DQIs [25,26,27,28,29,30,31,34,35,38,40,42,43,44,45,47,48] have been evaluated to some extent, with only two DQIs [40,44] having previously been applied to an IBD cohort. This is detailed further in Table 3.

### 3.4. DQI Meeting OptimaL Criteria for an IBD Population

No DQI included all optimal criteria for an IBD-specific DQI. The Dietary Guideline Index 2013 (DGI-2013) [44] most closely met recommendations, however, it only partially assessed the variety domain and did not use nonlinear scoring. This was followed by the Dutch Healthy Diet Index-2015 (DHD Index-2015) [40], the Planetary Health Diet Index (PHDI) [29] and the Healthy Eating Index for Australian Adults (HEIFA-2013) [42], that lacked in areas of variety and balance domain assessment, scoring structure, valuation and/or weighting.

### 3.5. Evaluation of DQIs

As detailed in Table 3, overall, 71% (17/24) of DQIs had undergone evaluation assessment, including validation, either by its original article or a separate evaluation study. Common validation methods performed included construct validity (*n* = 13) [25,26,28,29,30,31,34,38,40,48,57,63,64,70], criterion validity (*n* = 7) [28,29,30,38,48,55,64,68] and content validity (*n* = 3) [30,34,43]. Few DQIs were evaluated for reliability (n = 6) [25,29,42,57,60,70] or reproducibility (*n* = 1) [31].

Various adult populations were utilised for evaluation, with sample sizes ranging from 96–149,975 participants. Of the four DQIs most closely meeting the adapted optimal DQI criteria, all were validated in Western populations, including Australia [42,44], the Netherlands [40] and Brazil [29]. In addition to diet quality measurement, common health outcomes that were assessed against DQI scores included anthropometry (e.g., BMI, waist circumference, weight change) (*n* = 12) [26,28,30,34,40,41,44,48,63,64,67,72], biomarkers (e.g., lipid studies, blood glucose, plasma carotenoid concentration) (*n* = 7) [30,48,55,58,64,67,69], non-communicable disease risk, incidence and/or prevalence (e.g., type 2 diabetes, cardiovascular disease) (*n* = 6) [28,32,33,38,59,67,75] and mortality (*n* = 4) [36,38,73,75].

No DQI was validated to assess an IBD population. Two DQI were applied to FFQ from IBD participants, the DGI-2013 was applied to UC participants with ileoanal pouch [65] while the DHD Index-2015 was applied to participants with IBD or irritable bowel syndrome [66]. Another two DQI, the DDS and Australian Recommended Food Score (ARFS) were used with concurrent assessment of gut microbiota composition [56,62].

## 4. Discussion

This narrative review identified 24 current DQIs [25,26,27,28,29,30,31,32,33,34,35,36,37,38,39,40,41,42,43,44,45,46,47] and assessed their suitability for use with an IBD population against adapted optimal DQI criteria [14]. No DQI included all optimal criteria for an IBD-specific DQI. Most included adequacy and moderation dimension assessment, however, none sufficiently assessed all four dimensions (adequacy, moderation, variety, balance). The DQIs were heterogeneous across all other optimal criteria and had undergone varying extent of evaluation. No DQI was developed specifically for or validated in an IBD population. The DGI-2013 best met the criteria recommendations, followed by the DHD Index-2015, PHDI and HEIFA-2013. The DGI-2013 and DHD Index-2015 have been previously applied to an IBD population.

From an Australian general population context, two systematic reviews similarly found that no DQI met all optimal DQI criteria, with similarities and differences in construction, scoring and evaluation across all DQIs [16,17]. Acknowledging differences in eligibility criteria, consistent with our review, the DGI-2013 and HEIFA-2013 were identified by these systematic reviews as top performing against their own DQI criteria [16,17].

This review observed heterogeneity in all construction components of included DQIs. Diet quality indices outlined differing criteria for measuring foods, food groups and nutrient intake, for example, measuring “total vegetable” vs. “green vegetable” and “orange vegetable” intakes separately, and placed different valuation and weighting methods on these components based on the dietary guidelines and nutrition literature used to build the DQI. In addition, DQIs were built for and applied to differing dietary assessment methods (e.g., FFQ vs. 24 h Recall), with six developed for the specific assessment method they were applied to, such as the DDS developed for the EPIC-Norfolk FFQ. Further, there is increasing interest in use of digital assessment tools and artificial intelligence for dietary assessment. This requires consideration of how this data is captured (e.g., weighed food record vs. FFQ vs. food images) and analysed to determine how and if DQIs could be integrated into these existing platforms and tools [76,77,78]. Hence, for practicality, considering what and how dietary components are measured in a DQI (e.g., serve sizes, grams/day or % of energy) and whether a chosen dietary assessment method adequately captures these data is important for accurate application of a chosen DQI.

Acknowledging that no current DQI meets all optimal criteria for an IBD-specific DQI, it is ideal to select a DQI that meets the most optimal criteria that is appropriate for a chosen study design and reflects current population-based dietary guidelines or dietary pattern literature. For example, an Australian-based feeding trial where food provided is based upon the 2013 Australian Guide to Healthy Eating principles and diet is assessed according to Australian Dietary Guideline serving sizes should use the DGI-2013. This is particularly important when assessing change in dietary intervention trials as it has been demonstrated that DQIs are responsive to measuring dietary change over time where the DQI reflects the dietary pattern implemented in the intervention trial [79].

Further, it is important that the chosen DQI has been evaluated for use in assessing health outcomes of interest [80]. While 71% of DQIs in this review had undergone some form of evaluation, each assessed against different health outcomes (e.g., anthropometry, biomarkers, disease development risk) and none were validated to assess gut-specific health outcomes or outcomes relevant to an IBD population such as clinical and endoscopic disease activity. However, two DQI (ARFS and DDS) have been used with concurrent assessment of gut microbiota and another two DQI (DGI-2013 and DHD Index-2015) had been applied to an IBD population. Assessment of diet quality in IBD is increasingly emergent. In a systematic review assessing diet quality and IBD in adults [18], only one study utilised the Healthy Eating Index-2015 (HEI-2015), the previous version of the Healthy Eating Index-2020 (HEI-2020) included in our review. Of note, there are no scoring or component changes between the HEI-2015 and HEI-2020; instead, it was reviewed to reflect the current 2020–2025 Dietary Guidelines for Americans. With rapidly evolving diet research, food systems and population-based dietary guidelines, it is important to consider the currentness of a DQI to ensure it remains accurate and relevant. The remaining studies in the systematic review utilised DQI that were MED-based, lacking assessment of all food groups or nutrient-focused [18]. No evaluation of any DQI’s suitability or validity for an IBD population was undertaken. Other emerging works have utilised various DQI to assess the inflammatory potential of diet and risk of IBD. Most were primarily nutrient-based, assessing nutrients and random food (e.g., onion, rosemary, pizza) intakes, and were therefore limited in their ability to assess actual diet quality [81,82,83]. Others were DQIs developed for scoring food product nutrition labels [84] or for other diseases such as the cardioprotective diet score [85]. Recently, the dietary index for gut microbiota (DI-GM) has been developed specifically to assess dietary composition that aligns with gut microbiota diversity [86]. The DI-GM was excluded in this review as it does not capture assessment of all food groups, assessing 14 select foods (e.g., chickpeas, cranberries, red meat) chosen for their association with α-diversity and β-diversity indices and changes in specific defined bacteria; however, has been evaluated for construct validity, with the authors postulating the need for further evaluation of its utility for practice [86]. Few DQIs have been applied to therapeutic intervention diets for IBD. Lewis et al. [87] and Haskey et al. [88] used the HEI-2015 to assess diet quality of participants with IBD randomised to follow the Specific Carbohydrate Diet or MED, or Canadian Habitual Diet or MED, respectively, for 12 weeks. The HEI-2015, as previously discussed, is identical to the HEI-2020, which performed reasonably in our review but meets fewer optimal DQI criteria than other globally-applicable DQIs. Further investigation on the validity of current, relevant DQIs to appropriately assess diet quality when investigating gut-specific health outcomes is needed, including consideration of the appropriateness of markers used for microbial diversity and IBD.

In clinical practice, use of a DQI when assessing dietary intake could be considered. If used across multiple timepoints of clinical review, a DQI could provide insight into change in diet quality over time and may measure the impact of and adherence to dietary counselling [79]. However, practically, in these time-limited settings, an optimal DQI meeting recommendations for an IBD-specific DQI may be too time-intensive and with detailed scoring systems. Therefore, it would be important to consider the utility of DQI that meet fewer optimal criteria for an IBD-specific DQI but are more time efficient, or alternative dietary screeners and short tools not included in this review, such as the short Diet Quality Screener [89], REAP-S Dietary Screener Version 2 [90] or Eetscore [91], that may be a more appropriate methods for diet quality assessment in this setting.

This is the first known review to investigate if a suitable DQI for the adult IBD population exists, further strengthened by adaptation of known defined optimal criteria for DQI construction to an IBD population. This provides guidance for IBD diet researchers in selecting appropriate DQI to assess therapeutic IBD diets. Known limitations of a narrative review were minimised by applying systematic literature search strategies including screening of reference lists for further eligible articles. Without specific, defined dietary recommendations for IBD, this review cannot conclusively determine the appropriateness of a DQI for an IBD population. DQIs meeting most optimal criteria for an IBD-specific DQI assessed at minimum all five key food groups as current IBD dietary guidelines suggest. However, assessment for evolving dietary components postulated as important in IBD was not undertaken. Regardless of this lack of dietary recommendations, no DQI has been validated for an IBD population, posing opportunity for development of a validated, IBD-specific DQI.

## 5. Conclusions

Despite the emerging importance of assessing diet quality in IBD, this review identified that no existing DQI meets all recommended optimal criteria for an IBD-specific DQI. The DGI-2013 meets the most criteria, lacking in variety domain assessment and nonlinear scoring; this is followed by the DHD Index-2015, PHDI and HEIFA-2013, which to differing extents lack in variety and balance domain assessment, scoring structure, valuation and/or weighting. Hence, these DQIs, depending on country, study design and dietary assessment method, may be most appropriate for an IBD population. With no existing DQI validated for an IBD population, further research is required to ascertain an appropriate, validated IBD-specific DQI.

## Figures and Tables

**Table 1:** Diet quality indices applicable to adults with inflammatory bowel disease, published between 2014–2025.

Diet Quality Index	Country	Diet Quality Index Theoretical Framework *
Australian Diet Quality Score (ADQS) [33]	Australia	**Dietary Guideline**: Australian Dietary Guidelines and NRVs
Australian Recommended Food Score (ARFS) [31]	Australia	**Dietary Guideline**: Australian Dietary Guidelines (2013) **Reference Tool/s**: Recommended Food Score (1997)
Comprehensive Diet Quality Index (cDQI) [36]	United States	**Dietary Pattern/Literature**: WCRF/AICR Third Expert Report [49], GBD and NutriCoDE evidence review [50] **Reference Tool/s**: Plant-based Diet Index (2016)
Chinese Healthy Eating Index (CHEI) [47]	China	**Dietary Guideline**: Dietary Guidelines for Chinese (2016) **Dietary Pattern/Literature**: Current dietary status across China and the evidenced association of identified components with relative health outcomes **Reference Tool/s**: Healthy Eating Index-2010
CSIRO Healthy Diet Score (CSIRO-HDS) [35]	Australia	**Dietary Guideline**: Australian Dietary Guidelines (2013) **Reference Tool/s**: Short Food Survey “Diet Score” (2017)
Dietary Diversity Score (DDS) [32]	United Kingdom	**Dietary Guideline**: Australian Guide to Healthy Eating (2013) and United States MyPlate (2016) **Dietary Pattern/Literature**: United Nation’s Food and Agriculture Organization food group classification guidance
Dietary Guidelines for Americans Adherence Index-2020 (DGAI-2020) [38]	Canada	**Dietary Guideline**: Dietary Guidelines for Americans (2020–2025) and USDA Food Patterns **Reference Tool/s**: Dietary Guidelines for Americans Adherence Index 2015
Dietary Guideline Index-2013 (DGI-2013) [44]	Australia	**Dietary Guideline**: Australian Dietary Guidelines (2013) and Australian Guide to Healthy Eating **Reference Tool/s**: Dietary Guideline Index (2008); Recommended Food Score (2000)
Dutch Healthy Diet-Index 2015 (DHD-Index 2015) [40]	Netherlands	**Dietary Guideline**: Dutch Dietary Guidelines (2015) **Reference Tool/s:** Dutch Healthy Diet Index (2012)
Eat Lancet Diet Index (ELD-I) [37]	France	**Dietary Pattern/Literature:** EAT-Lancet Dietary Pattern [51] **Reference Tool/s:** EAT-Lancet Dietary Index (2019)
Ethiopian Healthy Eating Index (Et-HEI) [25]	Ethiopia	**Dietary Guideline**: Ethiopian food-based dietary guidelines (2022) **Reference Tool/s**: Healthy Eating Index (2021)
Food Choices Score (FCS) [34]	Australia	**Dietary Guideline**: Australian NRVs (2006) and Australian Guide to Healthy Eating 2013 Draft (2011) **Dietary Pattern/Literature**: Food groups definitions by Grafenauer et al. (2013) [52]
Global Diet Quality Score (GDQS) [28]	International	**Dietary Pattern/Literature**: “scientific evidence regarding relations between different foods and health” (42 references dating 1997 to 2020) **Reference Tool/s**: Prime Diet Quality Score (2018)
Healthy Eating Index-2020 (HEI-2020) [43]	United States	**Dietary Guideline**: Dietary Guidelines for Americans (2020–2025) **Dietary Pattern/Literature**: USDA Dietary Patterns **Reference Tool/s**: Healthy Eating Index-2015
Healthy Eating Food Index-2019 (HEFI-2019) [27]	Canada	**Dietary Guideline**: Canada’s Food Guide (2019) **Dietary Pattern/Literature**: Canadian Community Health Survey-Nutrition (2015) **Reference Tool/s**: Canadian Healthy Eating Index 2007
Healthy Eating Index for Australian Adults-2013 (HEIFA-2013) [42]	Australia	**Dietary Guideline**: Australian Dietary Guidelines (2013) and Australian Guide to Healthy Eating
Mexican Diet Quality Index (MxDQI) [41]	Mexico	**Dietary Guideline**: Mexican Dietary Guidelines (2015) **Dietary Pattern/Literature**: WHO, GBD and NutriCoDE evidence review [50]
Planetary Health Diet Index (PHDI) [29]	Brazil	**Dietary Pattern/Literature:** EAT-Lancet Dietary Pattern [51]
Programme National Nutrition Santé-Guidelines Score 2 (PNNS-GS2) [30]	France	**Dietary Guideline:** French Dietary Guidelines (2017) and French Nutrition and Health Programme **Reference Tool/s:** National Nutrition Santé–guidelines score (2009)
Quality Eating Index (QEI) [48]	Indonesia	**Dietary Guideline**: Indonesian Food Based Dietary Guideline (2014) **Reference Tool/s**: Healthy Eating Index-2015
RESIDE Dietary Guideline Index (RDGI) [26]	Australia	**Dietary Guideline**: Australian Dietary Guidelines (2013) and Australian Guidelines to Reduce Health Risks from Drinking Alcohol (2009) **Dietary Pattern/Literature**: Australian Heart Association and the American Heart Association Recommendations **Reference Tool/s:** Dietary Guideline Index (2008); Dietary Guideline Index-2013
Taiwanese Healthy Index (T-HEI) [39]	Taiwan	**Dietary Guideline**: Taiwanese Daily Food Guide (2018) **Reference Tool/s**: Healthy Eating Index-2005
Vietnamese Healthy Eating Index (VHEI) [46]	Vietnam	**Dietary Guideline**: Vietnamese Food Based Dietary Guidelines (2016–2020)
World Index for Sustainability and Health (WISH) [45]	Vietnam	**Dietary Pattern/Literature**: EAT-Lancet Dietary Pattern [51], GBD study by Afshin et al. (2017) [53], WHO Recommendations, Environmental Impact research by Clark et al. (2019) [54]

**Footnotes.** * Diet quality index theoretic framework outlines the dietary guidelines, dietary pattern or nutrition literature used to develop a diet quality index and any previous reference tools (e.g., diet quality indices) used to develop the diet quality index. **Abbreviations.** AICR, American Institute for Cancer Research; CSIRO; Commonwealth Scientific and Industrial Research Organisation; DQI, diet quality index; GBD, Global Burden of Disease; NRV, nutrient reference value; NutriCoDE, Nutrition and Chronic Disease Expert Group; USDA, United States Department of Agriculture; WCRF, World Cancer Research Fund; WHO, World Health Organization.

**Table 2:** Diet quality indices meeting adapted optimal criteria recommendations for an inflammatory bowel disease-specific diet quality index.

Diet Quality Index	Dimensions				Dietary Assessment	Scoring			Aggregation and Evaluation			
	Adequacy	Moderation	Variety	Balance	Assessment Method	Scoring	Scoring Units	Cut-Point *	Valuation	Unequal Weight	Evaluation	Applied to an IBD Cohort
						Ordinal, Dichotomous, Metric		Normative, Percentile, Uniform, Group-Specific	Linear, Nonlinear			
ADQS	Y	Y	N	P	DQES v2 FFQ	Ordinal	Grams/day	Normative, Group-specific	Both	Y	N	N
ARFS	Y	P	Y	N	mAES FFQ	Dichotomous	times or ADG serves/(day/wk)	None	Linear	N	Y	N
cDQI	Y	Y	N	N	24 h Recall	Metric	oz. or cup eq/ 1000 kcal	Both, Uniform	Linear	N	N	N
CHEI	Y	Y	P	N	24 h Recall	Metric	DGC-2016 serves/ 1000 kcal	Normative, Uniform	Linear	Y	Y	N
CSIRO-HDS	Y	N	Y	N	SFS FFQ	Metric	ADG serves/(day/wk/month)	Normative, Group-specific	Linear	N	Y	N
DDS	N	N	Y	N	EPIC-Norfolk FFQ	Ordinal	serves/ (day/wk/month)	Not specified, Uniform	Linear	N	N	N
DGAI-2020	Y	Y	P	Y	24 h Recall	Metric	oz. or cup eq/ (day/wk)	Normative, Uniform	Both	N	Y	N
DGI-2013	Y	Y	P	Y	FFQ	Metric	ADG serves/day	Normative, Group-specific	Linear	N	Y	Y
DHD Index-2015	Y	Y	N	P	FFQ, 24 h Recall	Metric	Grams/day	Normative, Group-specific	Both	N	Y	Y
ELD-I	Y	Y	N	N	FFQ	Metric	Grams/day	Normative, Uniform	Linear	N	N	N
Et-HEI	Y	Y	N	N	24 h Recall	Metric	Grams/day	Normative, Uniform	Both	N	Y	N
FCS	Y	Y	N	N	24 h Recall, Diet History	Ordinal	Serves/day	Both, Uniform	Both	N	Y	N
GDQS	Y	Y	P	N	FFQ, 24 h Recall	Ordinal	Grams/day	Percentile, Uniform	Both	Y	Y	N
HEI-2020	Y	Y	N	P	Not specified	Metric	oz. or cup eq/ 1000 kcal	Normative, Group-specific	Linear	N	Y	N
HEFI-2019	N	N	N	Y	24 h Recall	Metric	CFG serve or kcal	Both, Uniform	Linear	Y	Y	N
HEIFA-2013	Y	Y	P	P	FFQ, Weighed Food Record	Ordinal	ADG serves/day, mmol/day	Normative, Group-specific	Linear	Y	Y	N
MxDQI	Y	Y	N	P	24 h Recall	Metric	MDG serves, mL or grams/2000 kcal	Normative, Group-specific	Linear	Y	N	N
PHDI	Y	Y	N	P	FFQ	Metric	intake kcal/ total day kcal	Normative, Group-specific	Both	Y	Y	N
PNNS-GS2	Y	Y	N	Y	24 h Recall	Ordinal	FDG serve or grams/(day/wk)	Normative, Uniform	Both	Y	Y	N
QEI	Y	Y	N	N	FFQ	Metric	IFBDG serve/day	Normative, Uniform	Both	Y	Y	N
RDGI	Y	Y	N	P	FFQ	Ordinal	Serve or cups/day, times/(wk/month)	Normative, Group-specific	Linear	N	Y	N
T-HEI	Y	Y	N	P	FFQ	Metric	TDFG serve/1000 kcal	Normative, Group-specific	Linear	Y	N	N
VHEI	Y	Y	N	N	24 h Recall	Metric	VFBDG serves/day	Normative, Uniform	Both	N	N	N
WISH	Y	Y	N	N	24 h Recall	Metric	Grams/day	Normative, Uniform	Both	N	Y	N

**Footnotes. Green highlight (Y)**, DQI met adapted optimal DQI criteria(16); **Yellow highlight (P)**, criteria partially met; **Red highlight (N)**, criteria not met. * Normative = evidence-derived recommendations, e.g., nutrient reference values, guideline serving quantities; Percentile = derived from study population (e.g., quartile intakes); Group-specific = age and gender-specific recommendations; Uniform = same criteria applied to entire cohort. **Abbreviations**. ADG, Australian Dietary Guidelines; CFG, Canada’s Food Guide; DGC, Dietary Guidelines of China; DQES v2, Dietary Questionnaire for Epidemiological Studies Version 2; DQI, diet quality index; eq, equivalents; FDG, French Dietary Guidelines; FFQ, food frequency questionnaire; IFBDG, Indonesian Food Based Dietary Guidelines; kcal, calories; mAES, modified Australian Eating Survey; MDG, Mexican Dietary Guidelines; SFS, Short Food Survey; TDFG, Taiwan Daily Food Guide; VFBDG, Vietnamese Food Based Dietary Guidelines; Wk, week.

**Table 3:** Evaluation status of current identified diet quality indices applicable to adults with inflammatory bowel disease and health outcomes diet quality indices have been used to assess.

DQI	Evaluated	Evaluation Type	Population/s Evaluated In	Health Outcomes Assessed by Applying DQI
ADQS	No		242 adults aged 18–75 years from Australia	NCD—Depression [33]
ARFS	Yes	Reproducibility Construct validity—against nutrient intake Criterion validity—against plasma carotenoid concentration	96 adults aged 30–75 years from Australia 99 adults aged 18–60 years from Australia	Diet quality [31] Biomarker—Plasma carotenoid concentration [55] **Gut-specific**: Butyrate-producing bacteria [56]
cDQI	No		36,825 adults aged ≥20 years from the United States	Mortality—all cause, CVD and cancer-specific [36]
CHEI	Yes	Reliability—internal consistency Construct validity—against 2016 Dietary Guidelines for Chinese and population dietary risk factors	14,584 individuals aged ≥2 years from China 12,473 adults aged ≥18 years from China	Diet quality [47,57] Biomarker—Hyperuricemia [58] NCD—Metabolic Syndrome [59]
CSIRO-HDS	Yes	Reliability Direct validation—against 24 h recall nutrient intake Indirect validation—against population estimated nutrient intake	145,975 adults aged ≥18 years from Australia	Diet quality [35,60,61]
DDS	No		23,238 adults aged 40–79 years from the EPIC-Norfolk Cohort	Diet Diversity [32] NCD—T2DM development risk [32] **Gut-specific**: Microbial α-diversity [62] * Microbial β-diversity [62] * Gut microbial community [62] *
DGAI-2020	Yes	Face validity Construct validity—against sociodemographic, lifestyle factors and nutrient intake Criterion validity—against Eat Lancet Reference Diet Score and Plant-based Dietary Index	12,323 adults aged 45–80 years and 14,026 adults aged ≥18 years from Canada	NCD—CVD incidence [38] Mortality—CVD-specific [38]
DGI-2013	Yes	Construct validity—against nutrient intake Criterion validity—against sociodemographic and cardiometabolic risk factors Convergent validity—against participant health-related characteristics	4082 adults aged 55–65 years from Australia 141 adults aged 50–80 years from Australia 2689 adults aged 26–32 years from Australia	Diet quality [44,63,64] Anthropometry—BMI [44,63], WC [64] Biomarkers—Insulin resistance, cholesterol studies [64] Health-related behaviours (e.g., smoking, activity) [44] **Gut-specific**: UC population with ileoanal pouch [65]
DHD Index-2015	Yes	Construct validity—against participant characteristics and DHD index-2012	885 adults aged 20–70 years from the Netherlands	Diet quality [40] Anthropometry—BMI [40] **Gut-specific**: IBD and IBS population [66]
ELD-I	No		29,210 adults from France	Diet quality [37] Environmental—Environmental indicators, food consumption [37] Health gain score [37]
Et-HEI	Yes	Reliability—internal consistency Construct validity—against Minimum Dietary Diversity Score for Women and nutrient adequacy	494 women aged 15–49 years from Ethiopia	Diet quality [25]
FCS	Yes	Content validity—against possible methodological weaknesses Construct validity—against energy-deficit models and weight-loss trial data Internal validity—against diet models, nutrient values and current dietary recommendations External validity—against food categories, energy and nutrient intakes	195 adults from Australia	Diet Quality [34] Anthropometry—Weight change [34]
GDQS	Yes	Construct validity—against nutrient intake, NCD risk factors, Minimum Dietary Diversity Score for Women and Alternative Healthy Eating Index—2010 Criterion validity—against nutrient adequacy and NCD risk outcomes	Data from 9 cross-sectional and cohort datasets (sample size 1593–56,321) of women of reproductive age in 10 African countries, China, India, Mexico, and the United States 7517 women aged 15–49 years from Mexico	Diet quality [28,67,68] Anthropometry—BMI, WC [28,67] Biomarkers—ferritin, folate, lipid studies, glucose, insulin [67] T2DM incidence [28] Biometrics [28] NCD—Metabolic syndrome [67]
HEI-2020 **	Yes	Content validity—against Dietary Guidelines for Americans 2020–2025	Not specified	Diet quality [43] Biomarkers—Systemic inflammatory biomarkers [69]
HEFI-2019	Yes	Reliability—internal consistency Construct validity—against Canada’s Food Guide 2019, and United States Healthy Eating Index-2015	20,103 individuals ≥2 years old from Canada	Diet quality [70]
HEIFA-2013	Yes	Reliability—internal consistency Validity—against nutrient intake, weighed food records and FFQ	100 students aged 18–34 years from Australia	Diet quality [42] Dietary energy density [71]
MxDQI	No		2400 adults aged 20–69 years from Mexico 2310 adults aged 20–69 years from Mexico	Diet quality [41] Anthropometry—BMI, WC [41] Socioeconomic status [41]
PHDI	Yes	Reliability—internal consistency Construct validity—against nutrient intakes and EAT-Lancet Diet, Brazilian Healthy Eating Index revised Criterion validity—against participant characteristics	14,779 adults aged 35–74 years from Brazil	Diet quality [29] Anthropometry—BMI, WC [72] Mortality—total (all-cause + cause-specific (e.g., CVD, cancer, respiratory disease, neurodegenerative disease infectious disease) [73] Environmental—Carbon footprint [29]
PNNS-GS2	Yes	Face validity—against expert opinion Content validity—against expert opinion Convergent construct validity—against sociodemographic and biological values Criterion validity—against PNNS-GS	80,965 adults aged ≥18 years from France	Diet quality [30] Anthropometry—BMI [30,74] Biomarkers—lipid studies, blood glucose [30] Clinical—Blood pressure [30] NCD—Risk of CVD and cancer [75] Mortality [75]
QEI	Yes	Construct validity—against BMI and lipid studies Criterion validity—against BMI	415 adults aged 18–64 years from Indonesia	Diet quality [48] Anthropometry—BMI [48] Biomarkers—lipid studies [48]
RDGI	Yes	Construct validity—Measures of agreement against Australian Dietary Guidelines, original RDGI	1811 adults aged 19–78 years from Australia	Diet quality [26] Anthropometry—BMI [26] Physical Activity [26] Self-rated health [26]
T-HEI	No		154 adults aged ≥65 years from Taiwan	Diet quality [39] Frailty/pre-frailty [39]
VHEI	No		8225 adults responsible for food preparation in a household from Vietnam	Diet quality [46]
WISH	Yes	Validity—against duplicate 24 h dietary recalls	396 adults aged 18–49 years old from Vietnam	Diet quality [45] Environmental—sustainability [45]

**Footnotes.** Grey shade, indicates no evaluation, * = has one adaptation made to original DDS, ** = nil scoring or component changes between 2015 and 2020 edition, 2015 edition previously validated. **Abbreviations.** BMI, body mass index; CVD, cardiovascular disease; IBD, inflammatory bowel disease; IBS, irritable bowel syndrome; NCD, non-communicable disease; T2DM, type 2 diabetes mellitus; UC, ulcerative colitis; WC, waist circumference.

## Data Availability

No new data were created for this review. Literature search results and data extracted from articles by the authors are presented in this article and Supplementary Materials.

## Supplementary Materials

The following supporting information can be downloaded at: https://www.mdpi.com/article/10.3390/nu17142343/s1, Table S1: Full search strategy performed in MEDLINE and EmCare databases on 16 May 2025; Table S2: Full inclusion and exclusion criteria used to determine suitability of articles for literature review during title and abstract, and full-text screening; Table S3: Broad outline of food groups and nutrients included within four dimensions of diet quality for included diet quality indices.
