# Supplementary material for: Looking Beyond Nutrients, How to Assess Diet Quality in an Inflammatory Bowel Disease Population—A Narrative Review
Source: Nutrients. 2025 Jul 17;17(14):2343. doi: 10.3390/nu17142343 (PMC12299158; doi:10.3390/nu17142343)
Supplement: Supplementary file 1 [file nutrients-17-02343-s001.zip › nutrients-3746711-supplementary.pdf]

Looking Beyond Nutrients, How to Assess Diet Quality in an Inflammatory Bowel Disease Population—A Narrative Review

Supplementary Materials

Supplementary Table S1*. Full search strategy performed in MEDLINE and EmCare databases on the 16th May 2025.*

#	Query	MEDLINE Results	EmCare Results
1	(("diet*" or "healthy eating pattern" or "nutri*" or "food") adj3 ("index" or "indic*" or "score" or "tool" or "metric*")).ti,ab,kf.	45,005	21,146
2	(("diet*" or "healthy eating pattern" or "nutri*" or "food") adj3 ("quality" or "inflammat*")).ti,ab,kf.	45,586	20,390
3	1 and 2	7,788	4,860
4	limit 3 to yr="2013 -Current"	6,954	4,362
5	limit 4 to english language	6,836	4,246
6	limit 5 to humans	4,912	4,046

Supplementary Table S2*. Full inclusion and exclusion criteria used to determine suitability of articles for literature review during title and abstract, and full-text screening.*

Inclusion	Exclusion
• English language<br>• Full-text articles<br>• Human studies<br>• DQI designed for adults aged ≥18 years<br>• Original article that developed or updated the DQI<br>• A priori DQI based on the most current nutritional guidelines or recommendations to reflect the latest nutrition science from 2013 onwards<br>• DQIs published from 2013 onwards<br>• Most current version of a particular DQI<br>• Food-based dietary index measuring constructs similar to food groups or principles reflected among current national dietary guidelines (e.g., Australian Dietary Guidelines, Dietary Guidelines for America, etc.)<br>• DQI that summarise the overall diet into a single value<br>• DQI that assess an individual's dietary intake and can be applied to individual-level dietary assessment methods (e.g., Food Frequency Questionnaire, 24-hour Recall, Diet History, Weighed Food Records)	• Non-English language<br>• Articles not available in full text<br>• Cellular or animal studies<br>• DQI designed for specific sub-populations including infants, children, adolescents, lactating or pregnant women or athletes<br>• Not the original article the DQI was developed or updated in<br>• Abstracts only, review, commentary, editorial, conference proceeding and/or theses papers<br>• A posteriori or empirically derived indices such as those using factor analysis (principal component analysis) or cluster analysis<br>• DQIs published earlier than 2013, or utilising national dietary guidelines published earlier than 2013<br>• Not the most current version of a particular DQI or not based on the most updated version of respective national dietary guidelines<br>• Nutrient-only dietary index or lacking assessment of one or more key food groups as reflected by dietary guidelines globally<br>• Lifestyle indices (including >2 score components on exercise, smoking etc.)<br>• DQI that are applied to dietary intake at a non-individual level (e.g., household level or food industry, including food service menus and food labelling)<br>• DQI developed for prevention of specific diseases excluding gastrointestinal diseases and/or inflammatory bowel disease (e.g., cardiovascular disease, diabetes, etc.)

Abbreviations. DQI, Diet Quality Index

Supplementary Table S3. *Broad outline of food groups and nutrients included within four dimensions of diet quality for included diet quality indices.*

DQI	Four dimensions of scoring diet quality			
	Adequacy	Moderation	Variety	Balance
Australian Diet Quality Score	**Positive linear:**<br>Fruit<br>Veg<br>Whole grains<br>Protein – seafood, nuts<br>**Non-linear:**<br>Protein – meat, poultry, eggs, soy<br>Dairy	**Negative linear:**<br>UPF - processed grains, extras foods	NA	Fats - unsaturated fats/total fat<br>Extras - extras kJ/total kJ
Australian Recommended Food Score	**Dichotomous:**<br>Fruit<br>Veg<br>Grains<br>Protein – seafood, eggs, legumes, nuts, soybean<br>Dairy<br>Water<br>Spread/sauces	**Dichotomous:**<br>Protein – red meat, poultry	Points allocated to different foods consumed within a food group	NA
Comprehensive Diet Quality Index	**Positive linear:**<br>Whole fruit<br>Veg<br>Whole grains<br>Protein – poultry, seafood, legumes, nuts<br>Dairy<br>Veg oils<br>Coffee/tea	**Negative linear:**<br>Fruit juice<br>White potatoes<br>Protein - red meat, processed meat, eggs<br>UPF – refined grains, SSB, sweets, desserts	NA	NA
Chinese Healthy Eating Index	**Positive linear:**<br>Fruit<br>Veg – total, dark<br>Grains - total, wholegrains, mixed beans, tubers<br>Protein – poultry, seafood, eggs, nuts, soybean<br>Dairy	**Negative linear:**<br>Protein - Red meat<br>Cooking oils<br>Sodium<br>Added sugars<br>Alcohol	Some assessment of broad grain, veg and animal product sub-groups (e.g., total veg and dark veg)	NA
Commonwealth Scientific and Industrial Research Organisation Healthy Diet Score	**Positive linear:**<br>Fruit<br>Veg<br>Grains<br>Protein – animal and plant<br>Dairy<br>Healthy fats<br>UPF – discretionary foods<br>Beverages	NA	Points allocated to different foods consumed within a food group during the specified timeframe.	NA
Dietary Diversity Score	NA	NA	Points allocated to different food group subtypes within food groups (Fruit, Veg, Grains, Meat and alternatives, Dairy)	NA

Dietary Guidelines for Americans Adherence Index 2020	**Positive linear:**<br>Fruit<br>Veg – dark green, red/orange, legumes, other<br>**Non-linear:**<br>Veg – starchy<br>Grains<br>Protein – animal and plant<br>Dairy	**Negative linear:**<br>Added sugar<br>Cholesterol<br>Sodium<br>Alcohol	Points allocated to number of fruit and veg subtype food groups consumed	Grains - % whole grains/% total grains, g dietary fibre/1000kcal<br>Fats - % total fat/% total energy, % saturated fat/% total energy<br>Dairy/Meat - % low-fat dairy and meat/% total dairy and meat
Dietary Guideline Index 2013	**Positive linear:**<br>Fruit<br>Veg<br>Grains<br>Protein – meat, poultry, seafood, eggs, legumes, nuts<br>Dairy<br>Beverages/water	**Negative linear:**<br>UPF - discretionary foods<br>Unsaturated spreads and oils<br>Added salt<br>Added sugar<br>Alcohol	Points allocated to proportion of food from each of 5 core food groups eaten at least one serve/week	Grains – wholegrain bread/white bread<br>Dairy - reduced fat milk/whole dairy<br>Meat - lean meats/total meats<br>Water – water/total fluids
Dutch Healthy Diet Index 2015	**Positive linear:**<br>Fruit<br>Veg<br>Wholegrains<br>Protein – seafood, legumes, nuts<br>Tea<br>**Non-linear:**<br>Dairy<br>Coffee	**Negative linear:**<br>Protein – Red and processed meat<br>UPF – SBS, fruit juice<br>Fats and Oils<br>Salt<br>Alcohol	NA	Grains – wholegrain/total grains<br>Fats – liquid cooking fats/solid cooking fats
Eat Lancet Diet Index	**Positive linear:**<br>Fruit<br>Veg<br>Wholegrains<br>Protein – legumes, nuts	**Negative linear:**<br>Potatoes and tubers<br>Protein – meat, poultry, seafood, eggs<br>Dairy<br>Fat – saturated and unsaturated<br>Added sugars	NA	NA
Ethiopian Healthy Eating Index	**Positive linear:**<br>Fruit<br>Veg<br>Wholegrains, roots and tubers<br>Protein – legumes, nuts<br>**Non-linear:**<br>Protein – meat poultry, seafood, eggs<br>Dairy<br>Fats and oils	**Negative linear:**<br>UPF – SSB<br>Added sugars<br>Salt<br>Alcohol	NA	NA
Food Choices Score	**Positive linear:**<br>Fruit<br>Protein - seafood<br>**Non-linear:**<br>Veg – 'free', starchy<br>Grains – Whole, non-whole	**Negative linear:**<br>Protein - Fatty meat<br>Dairy – medium and whole (>3.5% fat)<br>Unsaturated oils and margarine<br>UPF - Non-core foods and drinks	NA	NA

	Protein – meat, poultry, eggs, legumes, nuts<br>Dairy – low-fat (<3.5% fat)	**Non-linear:**<br>Alcohol		
Global Diet Quality Score	**Positive linear:**<br>Fruit – citrus, deep orange, other<br>Veg - dark leafy green, cruciferous, deep orange, other<br>Deep orange tubers<br>Wholegrains<br>Protein – game meat, poultry, seafood, eggs, legumes, nuts<br>Dairy – low-fat<br>Liquid oils<br>**Non-linear:**<br>Protein - red meat<br>Dairy – high-fat	**Negative linear:**<br>Fruit juice<br>White roots and tubers<br>Protein - processed meat<br>UPF – refined grains baked foods, sweets, ice-cream, SSB, purchased deep-fried foods	Some assessment of broad fruit, veg and animal product sub-groups (e.g., citrus fruit, deep orange fruit, other fruit)	NA
Healthy Eating Index-2020	**Positive linear:**<br>Fruit – total, whole<br>Veg – total, greens and beans<br>Wholegrains<br>Protein – total, seafood, plant<br>Dairy	**Negative linear:**<br>UPF - Refined grains<br>Sodium<br>Added sugar<br>Saturated fat	NA	Fat – unsaturated fatty acids/saturated fatty acids, % total energy from saturated fats<br>Sugar - % total energy from added sugars
Healthy Eating Food Index-2019	NA	NA	NA	**Positive linear:**<br>Fruit and veg - fruit and veg/total foods<br>Grains – wholegrains/total foods, wholegrains/total grains<br>Protein – protein foods/total foods, plant proteins/total protein foods<br>Water – water/total beverages<br>Fats – unsaturated/total saturated fats<br>**Negative linear:**<br>Fats – saturated/total energy<br>Added sugar – free sugar/total energy<br>Sodium – sodium/total energy
Healthy Eating Index for Australian Adults	**Positive linear:**<br>Fruit<br>Veg<br>Grain – total, wholegrains<br>Protein – meat, poultry, seafood, eggs, legumes, nuts, soybean<br>Dairy<br>Unsaturated oils	**Negative linear:**<br>UPF – discretionary foods<br>Saturated fat<br>Added sugar<br>Added salt<br>Alcohol	Points allocated to broad food group subtypes within fruit and veg food groups	Fat - % saturated/total energy<br>Sugar - % added sugars/total energy<br>Water - % water-based beverages/total beverages

Mexican Diet Quality Index	**Positive linear:**<br>Whole fruit<br>Veg<br>Wholegrains<br>Protein – poultry, seafood, eggs, legumes<br>Dairy – low-fat<br>Polyunsaturated fat	**Negative linear:**<br>Fruit juice<br>UPF - Refined grains<br>Protein – red and processed meat<br>Added sugars<br>Sodium<br>Saturated fat	NA	Fat - % polyunsaturated/total energy, % saturated/total energy<br>Sugar - % added sugars/total energy
Planetary Health Diet Index	**Positive linear:**<br>Fruit<br>Veg<br>Wholegrains<br>Protein – legumes, nuts<br>**Non-linear:**<br>Tubers and potatoes<br>Protein – seafood, eggs<br>Dairy<br>Vegetable oils	**Negative linear:**<br>Protein – red meat, poultry<br>Fat - animal fats<br>Added sugars	NA	Veg – dark green veg/total veg, red and orange veg/total veg
Programme National Nutrition Santé – guidelines score	**Positive linear:**<br>Fruit<br>Veg<br>Wholegrains<br>Protein - legumes<br>**Non-linear:**<br>Protein – seafood, nuts<br>Dairy	**Negative linear:**<br>Protein – red and processed meat<br>UPF – SSB, sugary foods<br>Added fat<br>Salt<br>Alcohol	NA	Meat – white ham/total processed meat<br>Fat – % added fat/total energy, % ALA-rich and olive oils/other oils, plant fat/animal fat<br>Sugar - % sugar foods/total energy
Quality Eating Index	**Positive linear:**<br>Fruit<br>Veg<br>**Non-linear:**<br>Grains – rice/noodles, root/tubers<br>Protein – animal-based, tempeh/tofu	**Negative linear:**<br>Grains – refined flour<br>Protein – processed protein sources<br>UPF – sweets<br>Salt<br>**Non-linear:**<br>Fat – fried or sauteed dishes	NA	NA
RESIDE dietary guideline index	**Positive linear:**<br>Fruit<br>Veg<br>Grains<br>Protein - fish<br>Dairy<br>Water-based beverages	**Negative linear:**<br>Protein – lean, red and processed meat<br>UPF – fried potatoes, pastries, fast foods, sweets, cakes and desserts, SSB<br>Salt<br>Alcohol	NA	Water - % water/total beverages
Taiwanese Healthy Index	**Positive linear:**<br>Fruit<br>Veg – total, dark or orange<br>Wholegrains<br>Nuts and seeds<br>Protein – total, plant, seafood<br>Dairy	**Negative linear:**<br>UPF - Refined grains<br>Saturated fats<br>Sodium<br>Alcohol	NA	Fat - % fatty acids/total energy, % saturated/total energy

Vietnamese Healthy Eating Index	**Positive linear:**<br>Fruit<br>Veg<br>**Non-linear:**<br>Grains<br>Protein – meat, seafood, egg, soybean<br>Dairy<br>Fats and oils	**Negative linear:**<br>UPF - sugar and sweets<br>Salt and sauces	NA	NA
World Index for Sustainability and Health	**Positive linear:**<br>Fruit<br>Veg<br>Wholegrains<br>Protein - legumes<br>**Non-linear:**<br>Protein – Seafood, nuts<br>Dairy<br>Unsaturated oils	**Negative linear:**<br>Protein – poultry, red meat, eggs<br>Saturated oils<br>Added sugars	NA	NA

Footnotes. Positive linear, defines where a score increases with increasing intake of a particular food component; Negative linear defines where a score decreases with increasing intake of a particular food component; Non-linear defines where a score increases with increasing intake of a particular food component until a specific threshold reached, above which the score decreases with increasing intake. **Abbreviations.** %, percentage; g, grams; NA, Not assessed; SSB, Sugar-sweetened beverages; Veg, Vegetables
